# Molecular Functional and Transcriptome Analysis of *Arabidopsis thaliana* Overexpression *BrBBX21* from Zicaitai (*Brassica rapa* var. *purpuraria*)

**DOI:** 10.3390/plants13233306

**Published:** 2024-11-25

**Authors:** Mei Fu, Mengting Lu, Juxian Guo, Shizheng Jiang, Imran Khan, Umer Karamat, Guihua Li

**Affiliations:** Guangdong Key Laboratory for New Technology Research of Vegetables, Vegetable Research Institute, Guangdong Academy of Agricultural Sciences, Guangzhou 510640, China; fumei@gdaas.cn (M.F.); lumengtting@163.com (M.L.); guojuxian@gdaas.cn (J.G.); jiangshizheng2021@163.com (S.J.); dh18006@yzu.edu.cn (I.K.)

**Keywords:** B-box, *BBX*, *Brassica rapa*, gene function, transcriptome, anthocyanins

## Abstract

B-box transcription factors (TFs) in plants are essential for circadian rhythm regulation, abiotic stress responses, hormonal signaling pathways, secondary metabolism, photomorphogenesis, and anthocyanin formation. Here, by blasting the *AtBBX21* gene sequence, we identified a total of 18 *BBX21* genes from five distinct *Brassica* species (*Arabidopsis thaliana*, *Brassica rapa*, *Brassica oleracea*, *Brassica napus*, and *Brassica juncea*). The *BrBBX21-1* gene is most closely linked to the *AtBBX21* gene based on phylogeny and protein sequence similarities. The *BrBBX21-1* gene, which encodes a polypeptide of 319 amino acids, was identified from Zicaitai (*Brassica rapa* ssp. *purpuraria*) and functionally characterized. *BrBBX21-1* was localized within the nucleus, and its overexpression in *Arabidopsis* augmented anthocyanin accumulation in both leaves and seeds. We further performed an RNA-seq analysis between the *BrBBX21-OE* and WT *A. thaliana* to identify the key regulators involved in anthocyanin accumulation. In detail, a total of 7583 genes demonstrated differential expression, comprising 4351 that were upregulated and 3232 that were downregulated. Out of 7583 DEGs, 81 F-box protein genes and 9 B-box protein genes were either up- or downregulated. Additionally, 7583 differentially expressed genes (DEGs) were associated with 109 KEGG pathways, notably including plant hormone signal transduction, the biosynthesis of secondary metabolites, metabolic pathways, glutathione metabolism, and starch and sucrose metabolism, which were considerably enriched. A transcriptome analysis led us to identify several structural genes, including *DFRA*, *GSTF12*, *UGT75C1*, *FLS1*, *CHI1*, *4CL3*, and *PAL1*, and transcription factors, MYB90, TT8, and HY5, that are regulated by the overexpression of the *BrBBX21-1* gene and involved in anthocyanin biosynthesis. Altogether, these findings demonstrate the beneficial regulatory function of *BrBBX21-1* in anthocyanin accumulation and offer valuable information about the basis for breeding superior *Brassica* crops.

## 1. Introduction

Zicaitai (*B. rapa* ssp. *purpuraria*), a local seasonal vegetable, also regarded as a variant of chinensis, is valued for its unique flavor and high nutrient content [1]. *Purpuraria* exhibits a purplish-red hue due to its elevated anthocyanidins, a category of bioactive antioxidants that mitigate cardiovascular and cerebrovascular illnesses while offering hepatic protection and several physiological advantages [2]. Because of these beneficial characteristics, it may be very beneficial for consumer health and agricultural profitability to create and grow enhanced *B. rapa* ssp. *purpuraria* varieties. Thus, identifying the genetic processes that underlie the production of anthocyanins in these plants could potentially aid in selective breeding for improved nutritional value.

The B-box domain protein, known as BBX, a specific category of zinc finger proteins, has garnered significant interest due to its involvement in regulating growth processes in plants [3]. Recently, discovery of the *BBX* gene families was effectively defined within the genomes of various crops, including *Vaccinium corymbosum* [4], *Dioscorea opposita* [5], *Glycine max* [6,7], *Vitis vinifera* [8], *Brassica rapa* [9], *Brassica napus* [9], *Brassica oleracea* [9], and *Dendrobium officinale* [10]. The biochemical, molecular, and physiological dimensions of the *BBX* family have undergone thorough investigation in *A. thaliana* [11]. B-box1 and B-box2 are two quite conserved domains, consisting of forty amino acids, found in most of the BBX proteins [11]. Moreover, a CCT domain, derivate from CONSTANS, CO-like, and TOC1, is also found in some BBX proteins [12]. According to their structure and function, *BBX* genes are classified into five distinctive sub-families (I–V), based on the presence of B-box and CCT domains [13]. Therefore, investigation of the *BBX* gene diversity could help us comprehend its functions.

Due to different environmental stresses, *BBX* plays a role in multiple growth processes [12]. For example, in *D. opposita*, *DoBBX2*-OE and *DoBBX8*-OE plants enhanced tuber development [5]. In tomato (*S. lycopersicum*), CBF-dependent cold resistance was positively stimulated due to *SlBBX17*-OE, which also stimulated the transcription of the *SlHY5* gene [14]. Moreover, the *BBX* genes from *D. officinale* exhibited increased expression under methyl jasmonate (MeJA) treatment, including *DoBBX17*, showing a significant 65-fold upregulation after 24 h [10]. Under low temperature and UV-B stress, the *MdBBX20* (*M. domestica*) gene regulated *MdMYB1* expression by activating *MdHY5* [15]. Under white light, the expression of *PpBBX16* (*P. pyrifolia*) was increased, and it exhibited a strong interaction with *PpHY5* to enhance the activity of *PpMYB10* [16]. According to [17], *BBX* genes can directly or indirectly interact with the genes that synthesize anthocyanins, therefore modifying the levels of anthocyanin production. When exposed to light, *A. thaliana* plants overexpressing the *BBX21*-*BBX23* genes upregulated the anthocyanin level as compared to the control (Col-0); in contrast, the accumulation of anthocyanins was adversely downregulated by *BBX24*, *BBX25*, and *BBX32* [16,18,19].

There are currently about 600 distinct kinds of anthocyanins known to exist, 6 of which are found in large quantities in plants [20]. Plant color variations are influenced by anthocyanin accumulation patterns [20], synthesized via the flavonoid pathway and governed by a sequence of regulatory elements and structural genes [21]. MYB-bHLH-WD40, a TF complex, controls the transcription of anthocyanin accumulation in different crops [22], such as PpMYB10 and PpMYB114 [23]. AtMYB75 (*PAP1*) and AtMYB90 (*PAP2*) in *A. thaliana* and tomatoes (*S. lycopersicum*) stimulate the structural gene expression involved in anthocyanin formation [24,25,26]. By controlling the biosynthesis of anthocyanins, SIMYB75 influences the quality of tomatoes (*S. lycopersicum*) [27]. Structural genes, such as early and late biosynthetic genes, encode diverse enzymes throughout the plant anthocyanin biosynthesis process [28]. Flavanone 3-hydroxylase (F3H), chalcone isomerase (CHI), and chalcone synthase (CHS) are the enzymes encoded by the early genes, while anthocyanidin synthase (ANS), dihydroflavonol 4-reductase (DFR), and flavonoid 3-O-glycosyltransferase (UFGT) are encoded by late biosynthetic genes [29,30]. In asparagus (*A. officinalis*), *CHS*, *DFR*, and *UFGT* gene expression is also controlled by the MYB-bHLH-WD40 complex [31]. Anthocyanin production is influenced by other TFs also, including BBX [32], bZIP [33], and NAC [34]. There is no functional validation regarding the role of *BBX* genes in anthocyanin accumulation in *B. rapa*.

In our study, we identified a total of eighteen *BBX* genes from five different species: *A. thaliana*, *B. rapa*, *B. napus*, *B. oleracea*, and *B. juncea*. Based on multiple sequence alignments, motifs, and phylogenetic analyses, we further decided to explore the functional regulations of the *BrBBX21-1* gene. *BrBBX21-1* overexpression in *A. thaliana* #1 and #2 resulted in darker-colored leaves and seeds. To investigate the underlying process of anthocyanin production provoked by the *BrBBX21* gene, we conducted an RNA-seq analysis comparing the WT (Col-0) and *BrBBX21-OE*. This analysis identified some structural genes associated with anthocyanin biosynthesis resulting from the overexpression of the *BrBBX21-1* gene. These findings enhanced our understanding of anthocyanin accumulation regulation in *B. rapa* and offer valuable breeding resources.

## 2. Results

### 2.1. Identification and Sequence Alignment of BBX21 Genes

**Figure 1:**
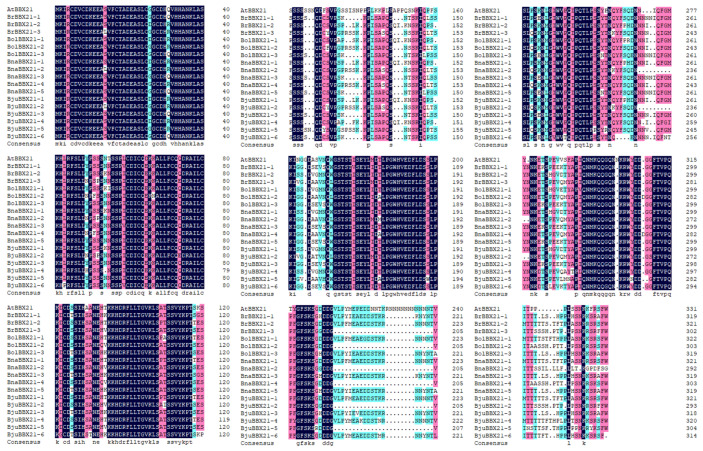
Multiple sequencing alignment of protein sequences of *BBX21* genes from *A. thaliana*, *B. rapa*, *B. oleracea*, *B. napus*, and *B. juncea* were utilized for alignment analysis.

We found a total of eighteen BBX21 proteins from five species, including *A. thaliana*, *B. rapa*, *B. oleracea*, *B. napus*, and *B. juncea* (Table S1). A multiple sequence alignment of all these selected proteins showed 89.05%, 88.33%, 84.40%, 84.35%, 88.33%, 88.04%, 79.64%, 83.92%, 76.39%, 81.29%, 73.46%, 86.72%, 88.02%, 72.83%, 79.19%, 80.07%, and 83.45% similarity with respect to AtBBX21 (Figure 1). This level of sequence conservation indicates that the *BBX21* gene is comparatively well-preserved in these species, which is probably due to its crucial function in anthocyanin and growth regulations by light and environmental response. BrBBX21-1 showed the highest similarity with AtBBX21 with 89.05% similarity, suggesting that this homolog might have conserved domains that play similar regulatory roles in photomorphogenic processes [35] and anthocyanin accumulation [36]. These results suggest that *BBX21* genes might continue to function consistently across species despite evolutionary divergence, which could provide cross-species insights into *BBX21*’s role in growth.

Three homologs of the *BBX21* gene have been identified in Zicaitai as *BrBBX21*-*1* (Bra003748), *BrBBX21*-*2* (Bra015835), and *BrBBX21*-*3* (Bra008204), encoding protein sequences of 319, 321, and 302 amino acids, with molecular weights of 35.44 kDa, 35.80 KDa, and 33.44 kDa, and theoretical pI values of 6.59, 6.54, and 7.54, respectively. Detailed information is given in Table 1. These physical–chemical characteristics suggest that *BrBBX21* homologs (*BrBBX21*-*1*, *BrBBX21*-*2*, and *BrBBX21*-*3*) may possess functional variations that may further impact their stability, location, and interactions with other proteins in the cell. Moreover, these homologs’ varying molecular weights and lengths of amino acid sequences imply that they may work in distinct but potentially complimentary processes linked to anthocyanin regulation.

### 2.2. Motif Analysis of BBX21

**Figure 2:**
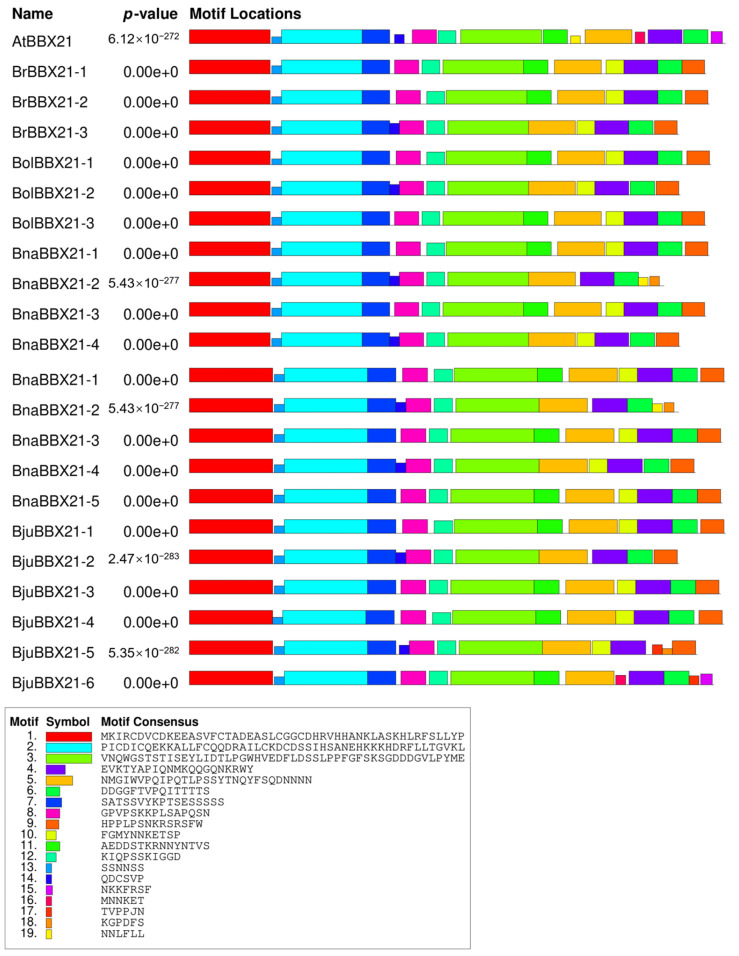
Motif analysis of protein sequences of *BBX21* genes from *A. thaliana*, *B. rapa*, *B. oleracea*, *B. napus*, and *B. juncea*.

Protein motif analysis is essential for discovering conserved areas that are pivotal in biological processes, including binding sites and enzymatic activity [37]. It facilitates the comprehension of protein structure–function interactions and can disclose evolutionary conservation, assisting in the formulation of tailored pharmaceuticals and cures [38,39]. In our study, the motif analysis exposed that the N-terminal region of the BBX21 proteins is enriched with highly conserved motif 1, motif 2, and motif 13, while the C-terminal region contains motif 4, motif 6, and motif 9 as the most conserved regions (Figure 2). The evolutionary conservation and crucial functional components of BBX21 proteins are highlighted by the motif analysis overall. By identifying conserved N-terminal and C-terminal motifs, this analysis highlighted the areas that are probably essential for preserving the protein’s functional integrity in different *Brassica* species, which may allow for more focused investigation into the function of *BBX21* genes in plant adaptability and resilience.

### 2.3. Domain and Phylogenetic Analysis

**Figure 3:**
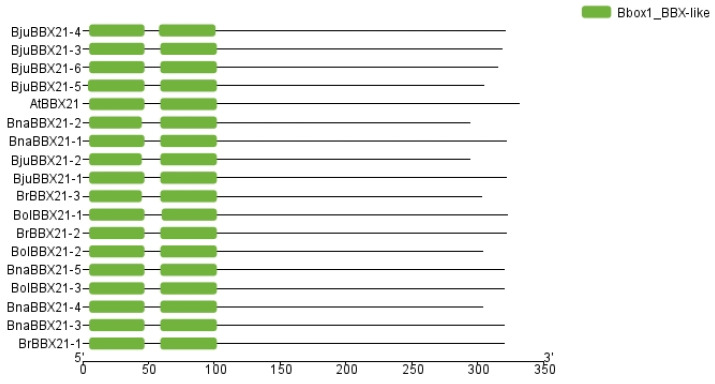
Domain analysis of BBX21 gene protein sequences from *A. thaliana*, *B. rapa*, *B. oleracea*, *B. napus*, and *B. juncea*.

The objective of a protein domain analysis is to distinguish functional and structural units within a protein, facilitating comprehension of its role, interactions, and evolutionary relationships [40]. We conducted domain analysis to examine the similar protein domains of BBX21 from various species (Figure 3). Our findings indicated that all selected BBX21 proteins possess a Bbox1_BBX-like domain, suggesting the conserved function of these proteins. This domain is indicative of BBX family proteins and highlights the critical role of *BBX21* genes in light-regulated development [41], photomorphogenesis [35], anthocyanin accumulation [36,42], and stress response [9,43,44], which seem to be evolutionarily conserved due to the domain’s intrinsic significance. It suggests that the Bbox1_BBX-like domain is indicative of a fundamental and conserved function that is essential across all BBX21 proteins.

**Figure 4:**
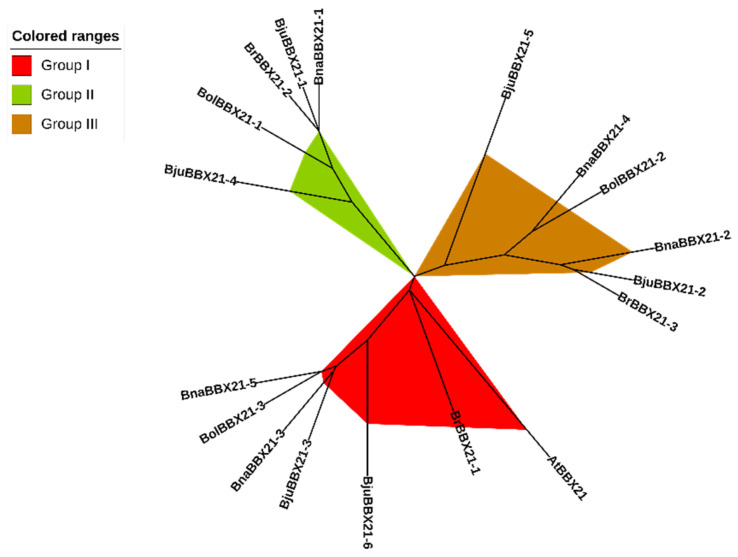
Phylogenetic analysis of BBX21 proteins from different species was conducted by MEGA-X version 10.2.4 software.

Phylogenetic analysis is an effective method for determining ancestral links and similarities in function between proteins from different species [45]. In this study, all identified BBX21 proteins from *A. thaliana*, *B. rapa*, *B. oleracea*, *B. napus*, and *B. juncea* were selected to construct a neighbor-joining (NJ) phylogenetic tree as a reference for AtBBX21. Based on the results, the tree was divided into three groups (group I-III), and we discovered that AtBBX21 has the highest similarities with BrBBX21-1, while BrBBX21-2 and BrBBX21-3 showed more resemblance to BjuBBX21-1 and BjuBBX21-2, respectively (Figure 4). Given the high similarity between AtBBX21 and BrBBX21-1, we chose the *BrBBX21-1* gene for further functional analysis to explore its role in *B. rapa* because it is likely to exhibit similar light-regulatory anthocyanin biosynthesis observed in *AtBBX21* [46,47]. The phylogenetic tree grouping of BBX21 proteins suggests that these proteins likely share a common ancestor and have diverged over time to adapt to species-specific needs and environmental conditions, but that some functional elements, such as light response and environmental adaptation, may be conserved despite divergence.

### 2.4. Overexpression of BrBBX21-1 Gene Promoted the Accumulation of Anthocyanins in A. thaliana

**Figure 5:**
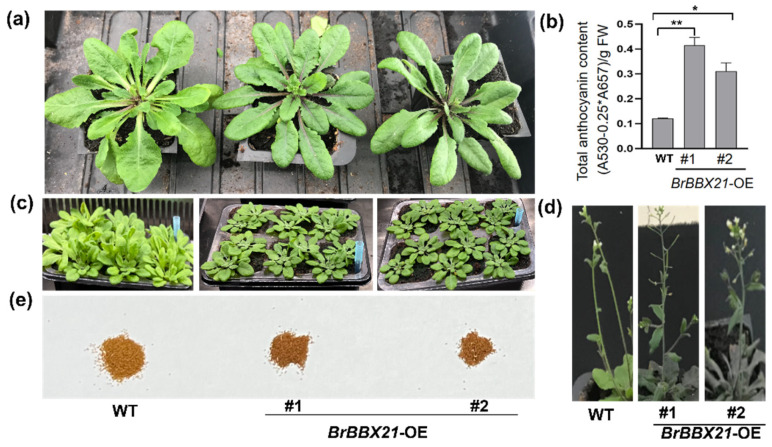
Phenotypic analysis of *A. thaliana* overexpressing *BrBBX21* gene: (**a**) phenotype of wild-type (WT) and *BrBBX21* overexpressed plants; (**b**) total anthocyanin content between WT and *BrBBX21*-*OE* plants; (**c**–**e**) in comparison to WT, the leaves and seeds of *BrBBX21-OE* lines exhibit a more purplish-red color. (*, *p* < 0.05; **, *p* < 0.01).

Different *BBX* genes have been discovered and studied as anthocyanin accumulators in numerous crops, such as the finding that ectopic expression of *OsBBX14* in *Arabidopsis* led to a significant enhancement in anthocyanin accumulation in its seedlings [48]. Overexpression of *MdBBX21* in *Arabidopsis* and apple calli under light stress enhanced anthocyanin accumulation [36]. The ectopic expression of PpBBX16 in *Arabidopsis* enhanced anthocyanin production in the hypocotyls and apices of flower stalks [49]. In our study, heterologous *BrBBX21*-*1* overexpressing *A. thaliana* #1 and #2 were grown for 4 weeks. The results showed that the leaves and seeds of *BrBBX21-OE* (#1 and #2) showed a darker color (Figure 5a,c,e), and the anthocyanin content was higher than that of the wild type (WT) (Figure 5b). During the flowering period, compared with the WT, the leaves of *BrBBX21-OE* lines showed a deeper purplish-red color (Figure 5d). These results highlighted that together with elevated anthocyanin levels, this visual alteration emphasizes *BrBBX21*-*1*’s crucial function in anthocyanin production. These results point to *BrBBX21*-*1* as a potential candidate for enhancing anthocyanin production in other commercially significant crops, with possible advantages for crop nutritional value, attractiveness, and environmental challenge adaptation.

### 2.5. Subcellular Localization of BrBBX21-1

**Figure 6:**
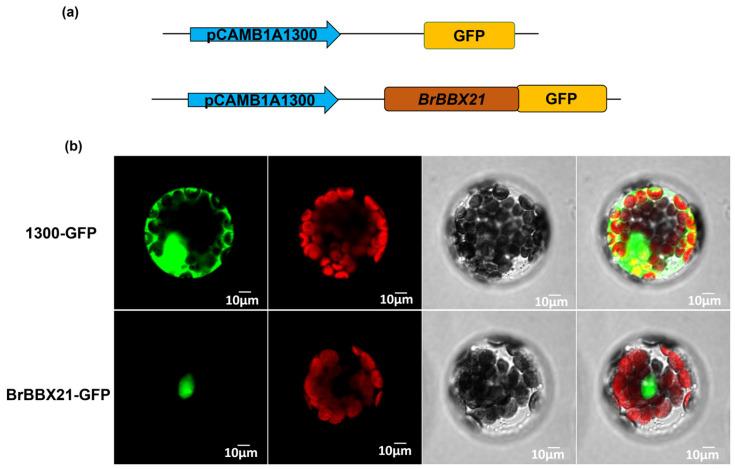
Subcellular localization of *BrBrBBX21*: (**a**) the construction of 1300: GFP (green fluorescent protein) and pCAMB1A1300: BrBrBBX21-GFP fusion proteins; (**b**) fluorescence images of 1300::GFP and pCAMB1A1300::BrBBX21-GFP fusion proteins.

To furnish more evidence for the prospective function of BrBBX21-1 in transcriptional regulation, the coding region of the *BrBBX21-1* gene and pCAMB1A1300-GFP vector were aligned to construct the BrBBX21-GFP expression vector. The vector was transformed into the protoplasts of *Arabidopsis* and the fluorescence signal was visualized with laser confocal microscopy (Figure 6). The results showed that the BrBBX21-1 protein was localized to the nucleus. Moreover, these findings provide evidence of the putative involvement of *BrBBX21-1* in transcriptional regulation, highlighting its capacity to modify genes associated with anthocyanin production and stress tolerance in *B. rapa* and other closely related species.

### 2.6. Transcriptome Analysis

**Figure 7:**
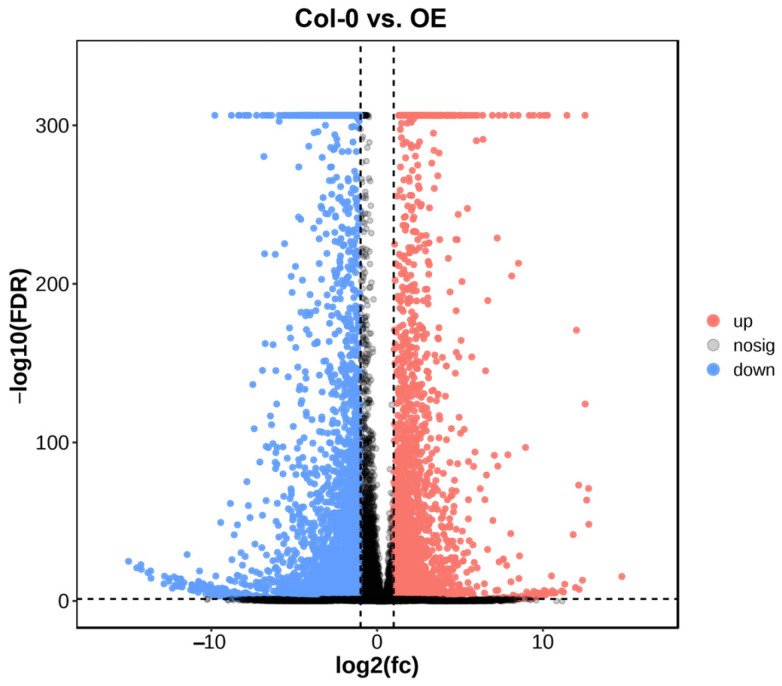
The volcano map illustrates the expression changes of all genes. Red represents the upregulated genes, whereas blue indicates the downregulated genes comparing the Col-0 and *BrBBX21-OE* samples.

The complicated, multi-step biochemical process of anthocyanin formation is controlled by a network of genes, numerous of which react to developmental signals or environmental stimuli [50,51,52]. To further explore the mechanism of anthocyanin production induced by the *BrBBX21-1* gene, we performed an RNA-seq analysis between the WT and *BrBBX21-OE*. Differentially expressed genes (DEGs) between control and transgenic plants were identified through statistical analyses. A total of 7583 genes exhibited deferential expressions, with 4351 and 3232 upregulated and downregulated genes, respectively (Figure 7, Table S2).

Typically, out of 7583 DEGs, a total of 81 F-box protein genes and 9 B-box protein genes were either upregulated or downregulated (Table S3). Key players in the ubiquitin–proteasome pathway, F-box proteins are frequently implicated in protein degradation, which influences light and stress responses [53,54,55]. By controlling upstream or downstream proteins in the route, they may also have an indirect impact on anthocyanin production. However, the presence of differentially expressed *B-box* genes refers to a possible interaction or feedback loop between *BrBBX21* and other *B-box* family members in controlling anthocyanin accumulation. These results demonstrate how intricately *BrBBX21* affects the transcriptional landscape and how it modulates pathways other than anthocyanin production, which may have an effect on several facets of plant growth, development, and stress tolerance.

### 2.7. GO and KEGG Enrichment Analyses

**Figure 8:**
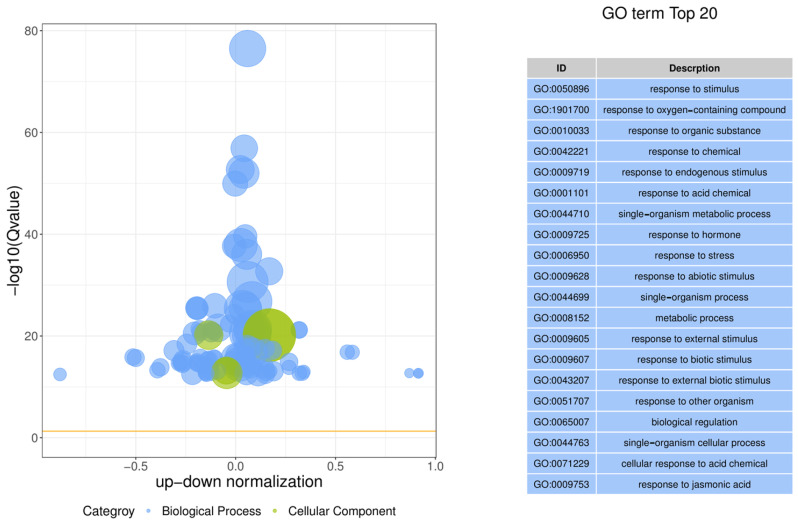
GO enrichment analysis of DEGs between Col-0 and *BrBBX21-OE* based on top twenty enriched terms, which include biological process (BP) and cellular component (CC).

We conducted Gene Ontology (GO) and Kyoto Encyclopedia of Genes and Genomes (KEGG) enrichment analyses to identify genes implicated in anthocyanin biosynthesis in *BrBBX21-OE*. We employed biological process (BP), molecular function (MF), and cellular component (CC) classifications as the foundation for the GO enrichment analysis to estimate the molecular function of these genes (Table S4). We found the top twenty enriched terms that are all related to the GO-BP annotation (Figure 8): response to stimulus (GO:0050896), response to oxygen-containing compound (GO:1901700), response to organic substances (GO:0010033), response to chemical (GO:0042221), response to endogenous stimulus (GO:0009719), response to acid chemical (GO:0001101), single-organism metabolic process (GO:0044710), response to hormone (GO:0009725), response to stress (GO:0006950), response to abiotic stimulus (GO:0009628), single-organism process (GO:0044699), metabolic process (GO:0008152), response to external stimulus (GO:0009605), response to biotic stimulus (GO:0009607), response to external biotic stimulus (GO:0043207), response to other organism (GO:0051707), biological regulation (GO:0065007), single-organism cellular process (GO:0044763), cellular response to acid chemical (GO:0071229), and response to jasmonic acid (GO:0009753). Detailed information of all enriched terms is given in Table S4.

**Figure 9:**
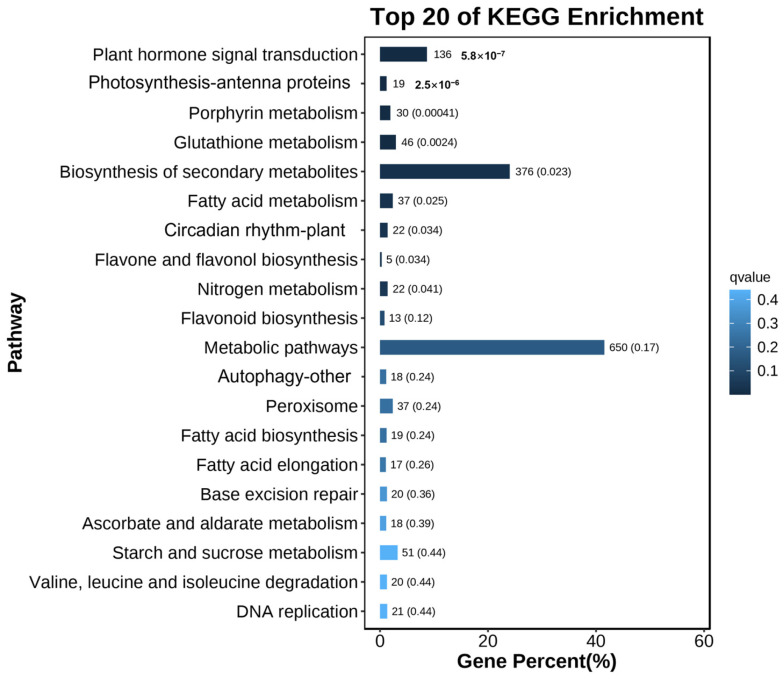
KEGG pathways of DEGs between Col-0 and *BrBBX21-OE*.

A functional enrichment of the 7583 differentially expressed genes (DEGs) was carried out to explore the Kyoto Encyclopedia of Genes and Genomes (KEGG) pathways. In detail, 7583 DEGs were mapped to 109 KEGG pathways (Table S5), emphasizing the significant regulatory impact of *BrBBX21* on many metabolic and signaling pathways (Figure 9), in which metabolic pathways, the biosynthesis of secondary metabolites, plant hormone signal transduction, starch and sucrose metabolism, and glutathione metabolism were significantly enriched (Figure 9). Significantly, pigment-related pathways were markedly enriched among the DEGs, offering critical insights into the mechanisms governing anthocyanin production in *BrBBX21-OE* plants. The observed rise in anthocyanin levels in *BrBBX21-OE* lines is consistent with the enrichment of these pigment-related and biosynthetic pathways, indicating that *BrBBX21* may interact with these pathways to promote anthocyanin production in addition to upregulating genes directly involved in anthocyanin biosynthesis. These results indicate that *BrBBX21* performed as a crucial regulatory center, coordinating several pathways to improve plant metabolism, stress tolerance, and pigment accumulation.

### 2.8. Identification and Validation of Anthocyanin-Related DEGs Regulated by BrBBX21-OE

**Figure 10:**
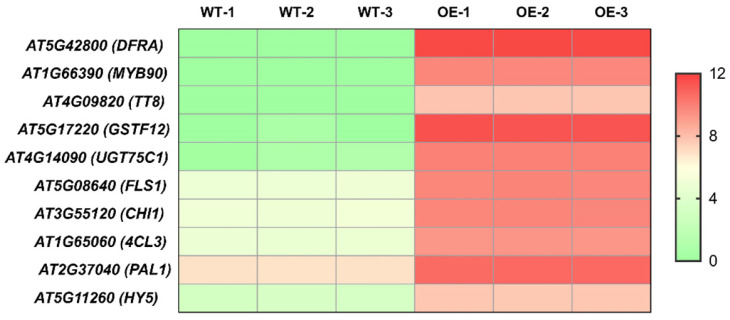
Expression of ten genes upregulated in *BrBBX21-OE* as compared to Col-0. To verify the accuracy of the transcriptome data, qRT-PCR was performed on eight genes related to anthocyanin synthesis. The results of these qRT-PCR tests aligned with the transcriptome findings (Figure 11). Expression levels of *MYB90*, *4CL3*, *FLS1*, *TT8*, *PAL1*, *DFRA*, *CHI1*, and *GSTF12* were upregulated in OE-BBX21 in both RNA-seq and qRT-PCR analyses, confirming the reliability of the transcriptome data.

Previous studies have identified the primary genes involved in the anthocyanin biosynthesis pathway expressed more during vegetative growth in red cabbage compared to green cabbage, leading to a variety of leaf hues [56]. Various colored mizuna, Chinese kale, *Arabidopsis*, and other plants exhibit integrated expression of *ANS*, *DFR*, *UFGT*, *F3′H*, and *F3H* [52,57,58,59]. In our study, we found several structural genes associated with anthocyanin biosynthesis due to overexpression of the *BrBBX21-1* gene, including *DFRA*, *GSTF12*, *UGT75C1*, *FLS1*, *CHI1*, *4CL3*, and *PAL1*, and transcriptome factors *MYB90*, *TT8*, and *HY5* (Figure 10), which aligns with the elevated anthocyanin phenotype of *BrBBX21-OE* in comparison to Col-0. Consequently, these specific DEGs probably play a role in the formation of differently colored leaves and seeds in *BrBBX21-OE* plants.

## 3. Discussion

Anthocyanins act not only as coloring agents but also as important antioxidants by enhancing plant resilience to different stressors [60,61]. Due to their elevated anthocyanin levels, *Brassica* vegetables, including mizuna (*B. rapa* var. *japonica*), headed Chinese cabbage (*B. rapa* ssp. *pekinensis*), ornamental cabbage (*B. oleracea* var. *acephala*), and broccoli (*B. oleracea* var. *italica*), have garnered great interest [62,63,64]. The anthocyanin metabolic pathway in *Brassica* crops has been linked to numerous TFs, such as bZIP, bHLH, MYB, MADS-box, etc. However, there is still no evidence about the role of BBX TFs [52,65,66].

Of the 32 *BBX* family members found in *Arabidopsis*, it has been found that *AtBBX20-25* and *AtBBX32* control the synthesis of anthocyanins [18,67,68,69,70,71,72]. We identified a total of eighteen *BBX21* genes across five different *Brassica* species by blasting the *AtBBX21* gene sequence. A protein sequence similarity and phylogenetic analysis revealed that *BrBBX21-1* shares the highest similarity with the *AtBBX21* gene, aligning with previous studies suggesting functional conservation among *BBX* genes [73,74,75]. To explore the role of *BBX* genes in anthocyanin accumulation in *B. rapa*, we generated transgenic *A. thaliana* lines overexpressing *BrBBX21-1*. The enhanced anthocyanin content observed in the leaves and seeds of these transgenic lines, relative to control lines, highlights a potential regulatory role for *BrBBX21-1* in anthocyanin biosynthesis. The result was similar to PpBBX16 [16], indicating that BBX proteins play an important role in anthocyanin biosynthesis.

*Arabidopsis* and other plants exhibited synchronized expressions of structural genes, including *DFR*, *ANS*, *F3H*, *F3′H*, and *UFGT*, to produce anthocyanins [57,58,59]. Dihydroquercetin, dihydromyricetin, and dihydrokaempferol possess a particular substrate preference in DFR found in various crops [59]. Furthermore, a crucial enzyme, ANS, facilitates the monochrome transformation into the colorful anthocyanins [58]. In addition, MYB90 and TT8 function as transcriptional activators in the MYB-bHLH-WD40 (MBW) complex [76], essential for activating genes like *UGT75C1*, which stabilizes anthocyanins through glycosylation [77], and *GSTF12*, which plays a role in anthocyanin transport [52,78]. Previous research revealed similar conclusions, identifying 23 structural genes particularly associated with anthocyanin biosynthesis, which include 3 *C4H*, 3 *PAL*, 1 *CHI*, 3 *CHS*, 3 *4CL*, 1 *DFR*, 3 *ANS*, 2 *F3′H*, 1 *F3H*, 2 *FLS*, and 2 *UFGT* genes [52]. In purple-stalked Chinese kale, the expression levels of seven structural genes were markedly elevated as compared to the green type [52]. Similarly, our work identified several structural genes associated with anthocyanin biosynthesis resulting from the overexpression of the *BrBBX21* gene, namely *DFRA*, *GSTF12*, *UGT75C1*, *FLS1*, *CHI1*, *4CL3*, and *PAL1*, and the transcription factors MYB90, TT8, and HY5. We speculate that phenotypic changes in the transgenic *A. thaliana* lines could be due to *BrBBX21-1*’s upregulation of key genes in the anthocyanin biosynthetic pathway, such as *DFRA*, *CHS*, and *F3H*. Additionally, *BrBBX21-1* may interact with transcription factors like MYB and bHLH, enhancing their activation of anthocyanin synthesis genes. BBX proteins have been found to stimulate target gene expression by interacting with a partner protein, such as HY5 [79]. HY5 is a transcriptional regulator of DNA binding and also regulates the transduction of light signals in plants [80]. It has been demonstrated in detail that the interaction of AtBBX21 and AtHY5 in *Arabidopsis* facilitates photomorphogenesis, including anthocyanin accumulation [18]. The *bbx20-bbx22* triple mutant exhibited a marked reduction in *HY5*-dependent gene expression and displayed phenotypic traits indicating reduced sensitivity to light, such as elongated hypocotyls and reduced anthocyanin accumulation [68]. Other studies have shown that PpBBX18 formed a heterodimer with PpHY5, in which PpHY5 bound to the G-box motif of *PpMYB10* and *PpBBX18* and provided trans-acting activity, thus inducing transcription of *PpMYB10* [16]. Transient co-expression of FaBBX24 and FaMYB5 in the cultivated strawberry ‘Xiaobai’ showed that co-expression strongly promoted the expression of *F3′H*, *4CL-2*, *TT12*, *AHA10*, and *ANR* and then increased the contents of anthocyanin and proanthocyanidin in the strawberry [81]. The previous identifications verified our conjectures; however, the specific processes require additional investigation. These findings extend our understanding of *BBX* gene function and provide novel insights into the molecular mechanisms underlying anthocyanin accumulation in *Brassica* species.

## 4. Materials and Methods

### 4.1. Identification of BBX21 Genes

By accessing the website http://www.brassicadb.cn/#/BLAST/ (accessed on 26 July 2024), the protein sequence of AtBBX21 was used as a query sequence to identify BBX21 peptides in various species. We found eighteen BBX21 protein sequences from *A. thaliana*, *B. rapa*, *B. oleracea*, *B. napus*, and *B. juncea*. Physical and chemical properties, such as molecular weight, isoelectric points, and the grand average of hydropathicity, were determined through the ExPASy website (http://web.expasy.org/protparam/, accessed on 26 July 2024).

### 4.2. Sequence Alignment, Motif Analysis, and Phylogenetic Analysis

Multiple sequence alignment was performed using the DNAMAN program, which facilitated the precise alignment of sequences to recognize preserved regions and sequence variants. The distribution of conserved motifs was evaluated using the MEME Suite (https://meme-suite.org/meme/tools/meme, accessed on 26 July 2024), which permitted a methodical investigation for statistically important motifs within aligned sequences. A phylogenetic tree was constructed with MEGA-X, employing the neighbor-joining method and 1000 bootstrap replications, to help in the assessment of evolutionary links among sequences. The resultant tree was displayed to illustrate evolutionary divergence and sequence grouping.

### 4.3. Construct and Transgenic Line Production

The 35S:BrBBX21-GFP construct was introduced into *A. thaliana* via Agrobacterium-mediated transformation, a common plant genetic modification approach. The floral dip method was used to infect flowering plants with Agrobacterium tumefaciens containing the plasmid comprising the 35S promoter driving the expression of the *BrBBX21-1* gene fused with green fluorescent protein (GFP). Infected plant seeds were harvested and seeded on Murashige and Skoog (MS) medium with 20 µg/mL hygromycin (Roche; www.roche.com) (accessed on 26 July 2024) to identify effectively transformed seedlings. After being transplanted to soil and developed under controlled greenhouse conditions, T3 homozygous lines were produced for future investigation. Two transgenic lines, *OE-BrBBX21* #1 and #2, were used for observation. Primers used in this experiment are given in Table S6.

### 4.4. Transient Expression of 35S:BrBBX21-GFP Protein

The complete CDS sequence of *BrBBX21-1* was amplified and inserted into the pCAMB1A1300-GFP vector using homologous recombination technology. The recombinant plasmid 35S:BrBBX21-GFP and the empty vector plasmid 35S:GFP were individually introduced into the *Arabidopsis* protoplasts. *Arabidopsis* protoplasts were prepared using 20 Arabidopsis plants about 3–4 weeks old. Fluorescence was observed using a confocal laser scanning microscope.

### 4.5. RNA Extraction, cDNA Library Construction, and Sequencing

*OE-BrBBX21*#1 transgenic and WT lines were used for the RNA seq analysis. Three biological replicates were taken from each group of samples, with each biological replicate consisting of a mixture of five plants. Total RNA was isolated from the leaves of *OE-BrBBX21*#1 transgenic and WT lines utilizing the Trizol reagent Kit (Invitrogen, Carlsbad, CA, USA). After grinding frozen leaves into a fine powder with a mortar and pestle, the lysis buffer was added to ensure homogeneity. After adding ethanol to help RNA attach to the column, the mixture was passed to the spin column. After centrifuging to bind RNA to the membrane, wash buffers were used to clean the column. Finally, centrifugation eluted pure RNA in RNase-free water. The quality of RNA was assessed using an Agilent 2100 Bioanalyzer (Agilent Technologies, Palo Alto, CA, USA). Paired-end cDNA libraries (WT-1, WT-2, WT-3, OE-1, OE-2, and OE-3) were generated utilizing a NEBNext Ultra RNA Library Prep Kit for Illumina (NEB #7530, New England Biolabs, Ipswich, MA, USA). The directions provided by the manufacturer were implemented for RNA-seq library preparation and sequencing, as previously described [82].

### 4.6. Pre-Processing of RNA Sequencing Data

Fastp (version 0.18.0) software was used to obtain clean, high-quality readings for analysis and assembly [83]. We used bowtie2 (version 2.2.8) to align the clean reads to the ribosome database [84]. After mapped readings discovered in the ribosomes were excluded, uncategorized reads were used for additional transcriptome analysis. High-quality readings were aligned to the Taro genome using HISAT2 2.4 using the HISAT2 program [85]. The fragments of transcript per kilobase per million mapped (FPKM) reads values were computed in order to assess the level of gene expression [86,87].

### 4.7. Gene Ontology (GO) and Kyoto Encyclopedia of Genes and Genomes (KEGG) Pathway Analysis

The Gene Ontology (GO) enrichment analysis of differentially expressed genes (DEGs) was performed utilizing the Cluster Profiler R package http://www.geneontology.org/ (accessed on 26 July 2024). This analysis identified the biological, molecular, and cellular components that are over-represented in the DEGs, offering insights into their potential roles in anthocyanin accumulation in *BrBBX21-OE*. The KEGG (Kyoto Encyclopedia of Genes and Genomes) pathway analysis was performed to elucidate the biological pathways associated with the differentially expressed genes. First, the gene identifiers from our dataset were converted to KEGG IDs using the KEGG database at http://www.genome.ad.jp/kegg/ (accessed on 26 July 2024), ensuring compatibility for analysis. The resulting list of KEGG IDs was then input into the KEGG PATHWAY Database at https://www.genome.jp/kegg/pathway.html (accessed on 26 July 2024) to retrieve relevant pathway information. Pathway diagrams were generated to visualize the interactions and relationships among the involved genes, providing insights into the metabolic processes and signaling pathways that may be influenced. To make additional analysis easier, DESeq2 was utilized to determine the DEGs between col-0 and *BrBBX21-OE*.

### 4.8. qRT-PCR Analysis

Total RNA was extracted using the Megan RNA Extraction Kit (Guangzhou Magen Biotechnology Co., Ltd. (Guangzhou, China)). The PCR primers used in this study are listed in Table S6, with the actin gene as the control. Each qRT-PCR reaction mixture comprised 0.3 µL of the specified primers, 1 µL of cDNA template, 3.4 µL of ddH_2_O, and 5 µL of 2 × ChamQ Universal SYBR qPCR Master Mix (Vazyme, Nanjing, China), making a final volume of 10 µL.

### 4.9. Data Analysis

Genes that fulfilled the requirement of |log2 fold change| > 1 and false discovery rate (FDR) < 0.05 were identified as differentially expressed genes (DEGs) in the transcriptome. For every experiment, three biological replicates were carried out, and the mean ± SD is used to present the data. Using *t*-tests with GraphPad Prism 8.0. lnk, statistical analyses were carried out, and significant differences were defined as *p* < 0.05.

## 5. Conclusions

In summary, we identified 18 *BBX* genes across five distinct species. Based on the strong similarity between the *BrBBX21* and *AtBBX21* genes, we investigated the molecular function of the *BrBBX21* gene. The overexpression of *BrBBX21* enhanced the purplish-red pigmentation of leaves and seeds and the manufacture of anthocyanins in *Arabidopsis*. Furthermore, we performed an RNA-Seq analysis between the WT (Col-0) and *BrBBX21-OE* to discover the candidate DEGs that are probably integrated with the *BrBBX21* gene to promote anthocyanin accumulation. These findings, while promising, are preliminary and require further experimental validation to confirm the functional role of *BrBBX21* in anthocyanin biosynthesis.

## Figures and Tables

**Figure 11:**
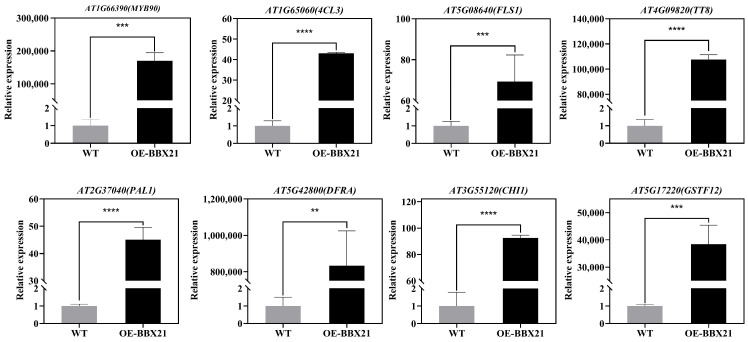
qRT-PCR verification of anthocyanin-related gene expression. Data are represented as relative expressions. Bars show means ± SD of biological replicate data. ** *p* < 0.01, *** *p* < 0.001, **** *p* < 0.0001.

**Table 1:** Detailed information of all BBX21 genes in five different *Brassica* species.

Gene ID	Gene Name	CDS/bp	Protein/aa	MW/kDa	pI
AT1G75540.1	*AtBBX21*	993	331	36.64	6.44
Bra003748	*BrBBX21-1*	957	319	35.44	6.59
Bra015835	*BrBBX21-2*	963	321	35.8	6.54
Bra008204	*BrBBX21-3*	906	302	33.44	7.54
BolC06g046810.2J	*BolBBX21-1*	966	322	35.78	7.11
BolC02g031890.2J	*BolBBX21-2*	909	303	33.43	6.44
BolC06g030760.2J	*BolBBX21-3*	957	319	35.41	6.26
BnA07g0299540.1	*BnaBBX21-1*	963	321	35.8	6.54
BnA02g0071300.1	*BnaBBX21-2*	879	293	32.09	6.44
BnA07g0288190.1	*BnaBBX21-3*	957	319	35.45	6.59
BnC04g0631670.1	*BnaBBX21-4*	909	303	33.34	6.69
BnC06g0758540.1	*BnaBBX21-5*	957	319	35.41	6.26
BjuVA07G41140.1	*BjuBBX21-1*	963	321	35.8	6.54
BjuVA02G24850.1	*BjuBBX21-2*	879	293	32.46	7.55
BjuVA07G28980.1	*BjuBBX21-3*	954	318	35.44	6.26
BjuVB03G48120.1	*BjuBBX21-4*	960	320	35.73	6.54
BjuVB05G57330.1	*BjuBBX21-5*	912	304	33.89	7.57
BjuVB06G41540.1	*BjuBBX21-6*	942	314	34.75	6.36

## Data Availability

The data presented in this study are available on request from the corresponding author.

## Supplementary Materials

The following supporting information can be downloaded at: https://www.mdpi.com/article/10.3390/plants13233306/s1, Table S1: List of all BBX21 protein sequences; Table S2: List of all identified DEGs; Table S3: List of all identified F-box and B-box TFs; Table S4: List of all GO enrichments; Table S5: List of all KEGG enrichments; Table S6: List of primers used in this study.
